# Evaluation of the immunogenicity and impact on the latent HIV-1 reservoir of a conserved region vaccine, MVA.HIVconsv, in antiretroviral therapy-treated subjects

**DOI:** 10.7448/IAS.20.1.21171

**Published:** 2017-05-22

**Authors:** Gemma Hancock, Sara Morón-López, Jakub Kopycinski, Maria C. Puertas, Eleni Giannoulatou, Annie Rose, Maria Salgado, Emma-Jo Hayton, Alison Crook, Catharine Morgan, Brian Angus, Fabian Chen, Hongbing Yang, Javier Martinez-Picado, Tomas Hanke, Lucy Dorrell

**Affiliations:** ^a^ Oxford NIHR Biomedical Research Centre, Nufﬁeld Department of Medicine, University of Oxford, Oxford, UK; ^b^ AIDS Research Institute IrsiCaixa, Institut d’Investigació en Cièncias de la Salut Germans Trias i Pujol, Universitat Autònoma de Barcelona, Badalona, Spain; ^c^ Computational Genomics Laboratory, Victor Chang Cardiac Research Institute, Darlinghurst, Australia; ^d^ University of New South Wales, Australia; ^e^ Oxfordshire Sexual Health Service, Oxford University Hospitals NHS Trust, Oxford, UK; ^f^ The Florey: Sexual Health, Royal Berkshire NHS Foundation Trust, Reading, UK; ^g^ Universitat de Vic – Universitat Central de Catalunya (UVic-UCC), Vic, Spain; ^h^ Institució Catalana de Recerca i Estudis Avançats (ICREA), Barcelona, Spain

**Keywords:** human immunodeficiency virus, therapeutic vaccine, viral inhibition assay, immunogen design, T cells, conservation, MVA, phase I trial

## Abstract

**Introduction**: Vaccines may be key components of a curative strategy for HIV-1. We investigated whether a novel immunogen, HIVconsv, designed to re-direct T cell responses to conserved viral epitopes, could impact the HIV-1 reservoir in chronic antiretroviral therapy (ART)-treated subjects when delivered by modified vaccinia virus Ankara (MVA).

**Methods**: Nineteen virologically suppressed individuals were randomized to receive vaccinations with MVA.HIVconsv (5.5 × 10^7^ plaque-forming units, pfu, n = 8; 2.2 × 10^8^ pfu, n = 7) or placebo (n = 4) at 0, 4 and 12 weeks. Magnitude, breadth and antiviral function of vaccine-induced T cells, cell-associated HIV-1 DNA in circulating CD4+ T cells and residual viremia in plasma were measured before and after vaccination.

**Results**: 90% of subjects completed the vaccine regimen; there were no serious vaccine-related adverse events. The magnitude of HIVconsv-specific IFN-*γ*-secreting T cells was not significantly boosted in vaccinees when compared with placebos in *ex vivo* Elispot assays, due to greater than expected variation in HIV-specific T cell responses in the latter during the observation period. Ex vivo CD8+ T cell viral inhibitory capacity was modest but significantly increased post-vaccination with MVA.HIVconsv at the higher dose (p = 0.004) and was positively correlated with the frequency of HIVconsv-specific CD8+ CD107+ IFN-*α*± T cells (r = 0.57, p = 0.01). Total HIV-1 DNA and residual viral load did not change significantly from baseline in any group.

**Conclusions**: Homologous prime-boost vaccination with MVA.HIVconsv was safe in HIV-positive ART-treated subjects but showed modest immunogenicity and did not significantly change the size of the viral reservoir. MVA.HIVconsv may be more effective when used in a heterologous prime-boost vaccination regimen and when combined with a latency-reversing agent.

**Clinical Trials Registration** NCT01024842

## Introduction

Therapeutic immunization has attracted renewed interest as a strategy to enhance immune-mediated clearance of persistent HIV-infected CD4+ T cells during long-term antiretroviral therapy (ART), with the goal of purging the latent viral reservoir to levels that are low enough to permit safe interruption of ART [1]. Recently, vaccine candidates have been tested either as the sole adjunct to ART or in combination with ART and latency-reversing agents (LRAs) as a means to “shock and kill” [2]. In the first scenario, it is envisaged that vaccination would induce the expansion of HIV-specific effector T cells with the capacity to kill virus-producing CD4+ T cells, thus reducing the frequency of latently infected cells and potentially delaying viral rebound during ART interruption [3,4]. Immunization with a human adenovirus vector expressing HIV Gag, a personalized vaccine based on autologous virus-pulsed dendritic cells or a Gag p24-based peptide vaccine, have shown modest yet statistically significant improvements in short-term virological control off ART [5–7]. In the second scenario, it is hypothesized that pharmacological reactivation of CD4+ T cells is needed to induce viral protein production and thus target the cell for immune recognition. This premise is supported by *in vitro* models of HIV-1 latency reversal; however, a poxvirus-vectored HIV-1 vaccine had only a transient effect on viral rebound whether tested alone or in combination with a candidate LRA, disulfiram in a phase I clinical trial [8,9].

These studies have been pivotal in demonstrating the safety and feasibility of testing new vaccines and LRAs and in assessing short-term effects on viral reservoir parameters in ART-treated subjects. However, more potent agents will be needed in order to achieve therapeutic benefit. Most HIV immunogens tested to date have been based on full-length HIV-1 proteins, an approach that may lead to preferential expansion of T cell clones targeting immunodominant and typically variable viral epitopes that have already escaped, recapitulating the ineffective responses induced by natural infection [3,10]. In the HIV-CORE 001 trial, we tested a conserved region immunogen, HIVconsv, vectored by modified vaccinia virus Ankara (MVA) in chronically infected ART-treated individuals. HIVconsv comprises 14 highly conserved regions of the viral proteome, assembled end-to-end as a chimeric protein and balanced for representation of the major clades, A-D. This vaccine had been shown to be immunogenic in mouse and macaque models prior to this trial [11,12].

By presenting the immune system with conserved HIV-1 sequences without the natural context of a full-length protein, we aimed to re-direct T cells towards viral epitopes that are vulnerable and typically sub-dominant.

## Methods

### Study participants

We conducted a randomized double-blind placebo-controlled study. This was approved by the Gene Therapy Advisory Committee (GTAC 165), the Medicines and Health Products Regulatory Agency (Eudract No. 2009-012662-31) and the Oxford University Hospitals NHS Trust. Written informed consent was obtained from all participants. Chronically HIV-infected adults aged 18–60 years who were virologically suppressed (<50 copies/ml) on combination ART and with CD4+ cell counts >350 cells/μl were randomly assigned in a 4:1 ratio to receive vaccine or placebo in two groups of ten, the first group receiving an MVA.HIVconsv dose of 5.5 × 10^7^ plaque-forming units (pfu) and the second, a dose of 2.2 × 10^8^ pfu. Subjects were HLA typed by amplification refractory mutation sequencing (ARMS)-PCR using sequence-specific primers.

### Randomization and masking

Study participants were randomly assigned to vaccine or placebo according to the study protocol using a computer-generated block randomization scheme, which was generated by an independent statistician and held securely with restricted access.

### Vaccine and vaccination schedule

**Figure 1.**
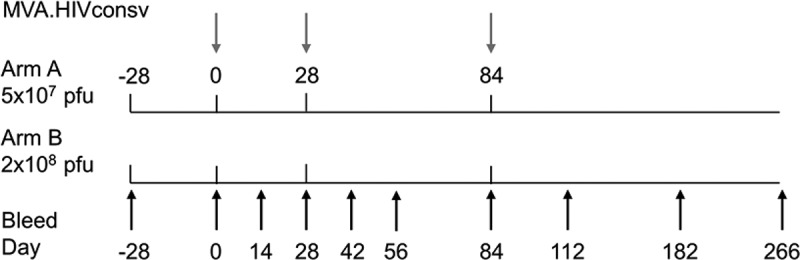
Vaccination Schedule. **Volunteers were screened up to 28 days before vaccination and randomized on day 0 to receive either 5.5** × **10^7^ pfu MVA.HIVconsv or placebo (Arm A, subjects 1–10, 4:1 ratio) and either 2.2** × **10^8^ pfu MVA.HIVconsv or placebo (Arm B, subjects 11–20, 4:1 ratio), by intramuscular injection on days 0, 28 and 84. Blood was sampled on the days indicated.**

The MVA.HIVconsv vaccine has been described previously [11]. It was manufactured and diluted in formulation buffer to 5 × 10^8^ pfu/ml by IDT Biologika GmbH, Germany. Formulation buffer was used as placebo. Vaccine and placebo vials were labelled by the manufacturer and stored at −80°C until use. The vaccine was thawed ≤30 min prior to administration and was given by intramuscular injection with a needle and syringe into the deltoid region of both arms (half of each dose per arm) on days 0, 28 and 84. Safety evaluations included reactogenicity symptoms recorded by the participants on diary cards following each vaccination, physical examination and monitoring of laboratory parameters. Blood samples were taken for safety and immunogenicity evaluations according to the study protocol schema shown (Figure 1).

### Peptides

HIVconsv peptides that span the HIVconsv immunogen (199 individual 15–17mer peptides overlapping by 11 amino acids) were pooled in a matrix format. The use of a peptide matrix enables deconvolution of the response and identification of individual peptide-specific responses using a two-step process [13]. First, fresh PBMCs from each time-point were tested against all matrix peptide pools in an IFN-γ Elispot assay. Individual HIVconsv peptides from pools giving a positive response were then retested in a second IFN-γ Elispot assay using cryopreserved PBMCs. Responses were either confirmed as true positives or discounted. A 4-dimensional matrix was designed using “Deconvolute This” (version 1.0 Roederer) and comprised 80 pools with 10 peptides per pool. 96-well plates containing working peptide pool stocks (4 µg/ml) were prepared in batches and stored at −80°C until required; final peptide concentration in the Elispot assay was 2 µg/ml.

### Ex vivo IFN-γ elispot assay

IFN-γ Elispot assays were performed as described previously [14]. The total HIVconsv-specific response was defined as the sum of the magnitude of all individual HIVconsv peptides retested in the second-round assay that elicited a response greater than the mean plus 2 standard deviations (SD) of negative control well values (100 SFU/million PBMCs).

### Intracellular cytokine assay

Intracellular cytokine secretion by antigen-specific T cell was analyzed using multiparameter flow cytometry. Briefly, cryopreserved PBMCs were thawed, washed and rested overnight in R10 medium at 37°C in a humidified incubator. Cells were stimulated with HIVconsv peptide pools (2 µg/ml), mock control and SEB (5 µg/ml) at 37°C for 6 h in the presence of Golgiplug, Golgistop (BD Biosciences) and CD107a BV421. Following viability and surface staining, cells were then fixed using BD cytofix/cytoperm solution (according to manufacturer’s instruction) and intracellularly stained using reagents as listed in Supplementary Table 1. At least 50,000 viable singlet CD3+ lymphocyte events were acquired using BD Fortessa X20. Data were analyzed using FlowJo v9.9.3 (FlowJo, US) and GraphPad Prism v7.0.

### Viral inhibition assay

The viral inhibition assay has been described in detail previously [15]. CD8+ T cells were isolated from cryopreserved PBMC by magnetic bead selection and used ex vivo without stimulation. The CCR5-tropic clade B laboratory-adapted HIV-1 isolate, BaL, was used for CD4+ T cell super-infection at a multiplicity of infection of 0.01. CD8+ T cell antiviral activity was reported as % inhibition.

### Quantification of HIV-1 proviral DNA in CD4+ T cells

CD4+ cells were isolated from thawed PBMC by magnetic bead selection. Lysed extracts from CD4+ T cells were used to measure total cell-associated HIV-1 DNA by ddPCR with primers and probes located in 5-LTR and Gag [16,17]. The RPP30 cellular gene was quantified in parallel to normalize sample input [18–20].

### Residual plasma viremia

To evaluate HIV-1 RNA below 50 copies/ml, 3–5 ml of plasma samples were ultracentrifuged and quantified using the Abbott Real-Time HIV-1 assay (Abbott Molecular Inc.), as previously described [21].

### Statistical analysis

In view of the inter-subject variation in baseline responses and response kinetics post-vaccination, HIVconsv-specific T cell IFN-γ responses were analyzed over time and according to group allocation using linear mixed effects models [22,23]. CD8+ T cell viral inhibitory activity and total HIV-1 DNA were analyzed over time and according to group by non-parametric tests using Graphpad Prism software (version 7.0). Censored matched pairs of ultrasensitive viral load were analyzed using the paired Prentice-Wilcoxon test (MiniTab).

## Results

### Study participants and vaccinations

**Figure 2.**
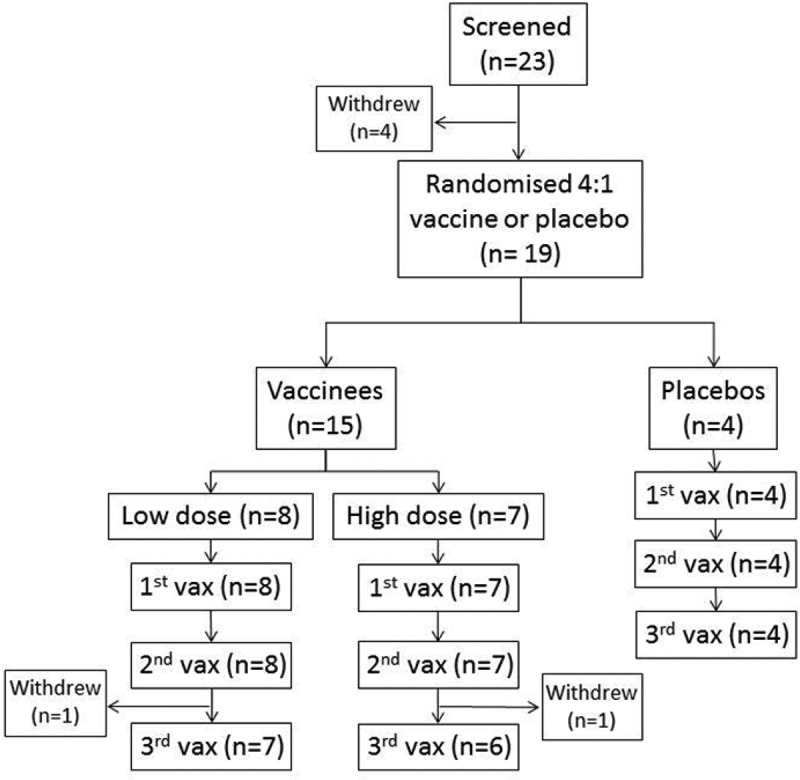
CONSORT flow diagram for recruitment and follow-up in the HIV-CORE 001 trial.

Nineteen subjects (14 men and 5 women) were enrolled in the study. Enrolment was stopped prior to reaching the planned 20 subjects due to slow recruitment. Vaccine and placebo recipients did not differ significantly in age, CD4+ cell count, CD4+ cell nadir, duration of ART or duration of diagnosed HIV infection at baseline. Volunteer characteristics are summarized in Table 1. All volunteers received their vaccinations as scheduled, except for one vaccinee in each of the low- and high-dose groups, who both withdrew after their second immunization (Figure 2).

**Table 1.** Clinical characteristics of trial participants

	All cohort n = 19	MVA-HIVconsv n = 15	Placebo n = 4	P Value
Male, n (%)	14 (70)	11 (73)	3 (75)	-
Age, years	46 (43–46)	45 (42–48)	52 (47–52)	0.24
ART duration, months	37 (24–58)	41 (25–60)	26 (22–36)	0.35
CD4+ count, cells/µl	630 (470–830)	580 (445–960)	635 (608–650)	0.56
CD4+ pre-ART nadir, cells/µl	170 (250–330)	160 (90–229)	255 (210–288)	0.10
Known protective HLA class I alleles (n)*	5	5	0	-

ART duration and CD4+ count values are median (IQR). *HLA-B*27, B51, B*5701/03, B*5801 and B*810.

### Vaccine safety

Vaccination was safe and well tolerated. The majority (14/15) of vaccine recipients experienced mild (n = 10) or moderate (n = 4) pain at the injection site and 2/15 developed a mild local reaction. Nine vaccinees experienced mild to moderate malaise, two reported mild headache and four reported “chills” after vaccination. All of these reactions resolved spontaneously within 48 h (Supplementary Table 2). Mild pain, headache and moderate malaise were also reported by each of 3/4 placebo recipients. No subject experienced a severe local or systemic reaction. A total of 86 adverse events were reported during following (66 in the vaccinees and 20 in the placebos); of these, 51 were grade 1, 27 were grade 2, five were grade 3; in three, the grading scale was not applicable (Supplementary Table 3). The grade 3 events occurring in vaccinees were all deemed to be unrelated to vaccination (n = 3, vomiting requiring hospitalization followed by development of a pulmonary abscess; pyelonephritis; gout). One placebo recipient developed Hodgkin’s lymphoma. No unexpected or vaccine-related serious adverse events occurred during the trial.

All patients remained virologically suppressed (HIV RNA <50 copies/ml) throughout follow up, apart from three subjects who were found to have detectable viraemia (69–1404 copies/ml) on a single occasion which was not confirmed on repeat testing. CD4 cell counts did not change significantly during follow up (p = 0.9, one-way ANOVA, Supplementary Figure 1)

### T cell responses to MVA.HIVconsv

**Figure 3.**
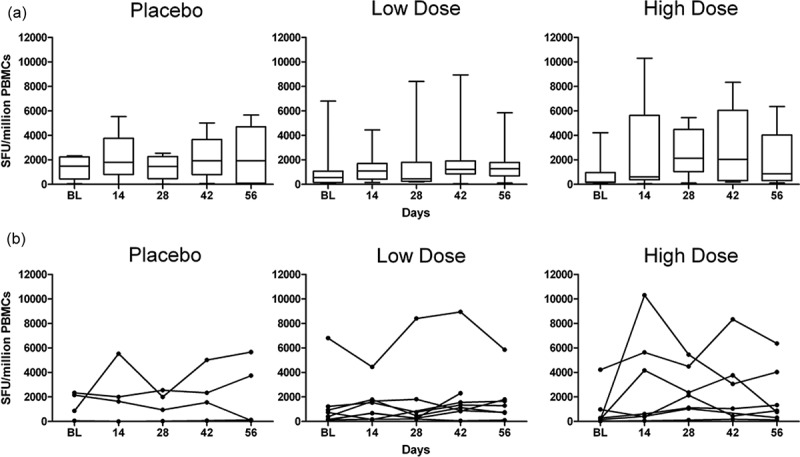
T cell immunogenicity of MVA.HIVconsv in volunteers over time. Total magnitude of T cell responses to the HIVconsv immunogen was assessed in ex vivo IFN-γ ELISPOT assays in (L to R) placebos, low dose vaccinees and high dose vaccinees shown as (a) box and whisker plot (median, IQR and range) and (b) line graphs (representing each subject). Total magnitude was calculated from the sum of all HIVconsv individual peptides tested in a confirmatory second round assay which gave a response above the mean plus 2* SD of negative control wells (100 SFU/million PBMCs, see Materials and Methods). BL – baseline.

**Figure 4.**
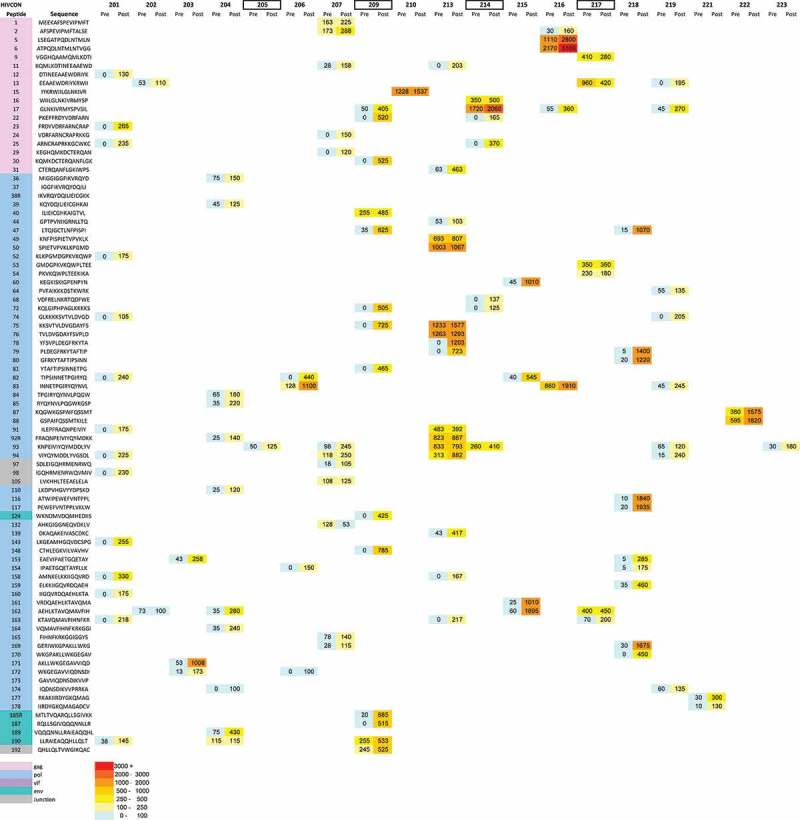
Breadth of pre- and post-vaccination responses to HIVconsv.**Location of confirmed positive peptides across the HIVconsv immunogen pre and post-vaccination (included if peptide was above 100 SFU/million PBMCs at any time-point after screening/day 0). Left-hand column shows peptide numbers according to antigenic origin: pink – gag; blue – pol; purple – vif; turquoise – env; grey – junctional peptide (15-mer peptides that span junctions between segments from different proteins or between segments from the same proteins yet from different clades). Colour and number in boxes indicate magnitude of response. Patient numbers outlined with a black box indicate placebo volunteers**.

IFN-γ Elispot assays with the 4-dimensional HIVconsv peptide matrix were performed using fresh PBMCs in all subjects at screening, day 0, day 14, day 56 and day 266 time-points, as a minimum. All deconvoluted matrix data up to day 56 were collated and individual peptide sets for re-testing were devised for each subject based on these data. The day 56 visit was chosen because the peak response was attained in the majority of subjects after two MVA vaccinations in other therapeutic vaccine trials [14,24]. The patient-specific peptide sets were then tested using cryopreserved PBMC from each time-point in a single IFN-γ Elispot assay. Pre-vaccination responses to HIVconsv were detected in 16/19 subjects, with a median frequency of 798 (range 110–6807) SFU/million PBMCs. Following vaccination, the peak response was not synchronized among the vaccinees, in contrast to previous studies of therapeutic vaccines encoding full-length HIV-1 proteins [14,25], and occurred between days 14 and 56. All 15 vaccinees had HIVconsv-specific responses above 100 SFU/million PBMCs at the peak of response and the median frequency was 1790 (range 180–10300) SFU/million PBMCs vs. 285 (range 110–6807) SFU/million PBMCs at baseline. However, responses among placebos also showed variation over time (Figure 3). In view of the inter-subject variation in baseline responses and response kinetics post-vaccination, linear mixed models were used to test for an interaction between HIVconsv responses over time and vaccination. The magnitude of responses increased significantly over time (p = 0.001) but differences between vaccinees and placebos were not statistically significant (p = 0.48) (Table 2A). IFN-γ responses to an influenza/EBV/CMV peptide pool remained constant during the study period, indicating that the variability in HIVconsv responses was not due to technical issues such as variation in assay conditions (Supplementary Figure 2). HIVconsv-specific responses in placebos were analyzed further by multiparameter flow cytometry. This confirmed the previously observed variation, not only in the frequency of single IFN-*γ*-positive cells (Supplementary Figure 3) but also in the frequency of polyfunctional T cells. Pre- and post-vaccination responses to HIVconsv were distributed evenly across the immunogen and increased in breadth over time (Figure 4, Supplementary Table 4), although the difference between vaccinees and placebos was statistically inseparable (Table 2B). Responses were mediated predominantly by CD8+ T cells (Supplementary Figure 4). There was a significant correlation between magnitudes of responses at baseline and at the peak of response in the vaccinees (r = 0.63, p < 0.0001; Supplementary Figure 5) suggesting that MVA.HIVconsv vaccinations had boosted pre-existing responses.

**Table 2.** Linear mixed model investigating the effect of MVA.HIVconsv vaccination on (A) magnitude and (B) breadth of responses over time

Variable	dF	F	p
**A**			
Intercept	644	17.2	0.00004
Time (days)*	644	3.3	0.00100
Group^§^	17	0.5	0.48300
**B**			
Intercept	70	28.1	0.000001
Time (days)*	70	2.90	0.007900
Group^§^	16	0.59	0.450000

Linear mixed-effects models were fitted to the data following a described model formulation [22] and computational framework [23] to investigate the effect of MVA.HIVconsv vaccination on the (A) magnitude of response and (B) breadth of response over time (Variable; time*) and between vaccines and placebos (Variable; group^§^). Breadth was defined as the number of positive peptides (response over 100 SFU/10^6^ PBMC) at each time point.

**Figure 5.**
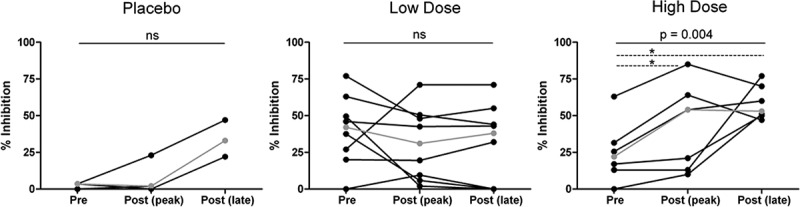
CD8+ T antiviral activity is increased following vaccination with a higher dose of MVA.HIVconsv. **CD8+ T cell-mediated inhibition of HIV-1_Bal_ in autologous CD4+ T cells (CD8+/CD4+ cell ratio = 1:1) was measured in all 19 trial participants at baseline (Screening or day 0), post-vaccination, concurrent with the peak response in the IFN-γ Elispot assay, and at a late post-vaccination time-point (day 182 or day 266) except one placebo recipient (pre-vaccination and peak only). This individual was therefore excluded from the statistical analysis. Grey line indicates median response. Asterisks indicate statistically significant differences in multiple comparisons analysis.**

CD8+ T cell antiviral activity was measured in all trial participants at one pre-and 2 post-vaccination time points, except for one of the placebo recipients for whom sample limitations permitted testing of two time-points only. The pre-vaccination median inhibitory response was 24% overall (3.5%, 42% and 22% in placebos, low-dose and high-dose vaccinees respectively) when measured at a CD4+/CD8+ T cell ratio of 1:1 (p = 0.057, Kruskal–Wallis test). At the peak of response post-vaccination, median inhibition was 4.6%, 12.5% and 54% in placebos, low-dose and high-dose vaccinees respectively. In the high-dose vaccinees there was a statistically significant increase in inhibition following vaccination with MVA.HIVconsv (p = 0.004, Friedman test; Figure 5), but no such trend was observed in the placebos or low-dose vaccinees (p = 0.19 and 0.29, respectively). Analysis was limited due to sample issues resulting in only 3 data points in the placebo group at peak. For this reason, we performed a 1-way ANOVA to increase statistical power when comparing inhibition longitudinally in vaccinees and placebos. A 1-way ANOVA of viral inhibition in high-dose and low-dose vaccine groups combined did not show any significant increases post vaccination.

**Figure 6.**
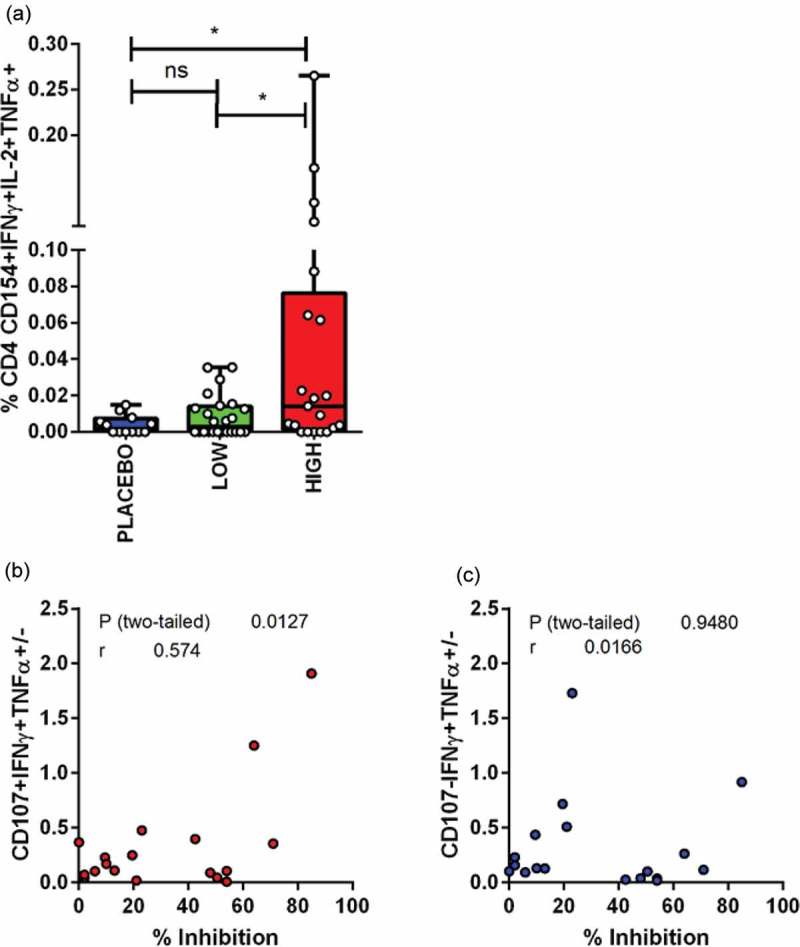
Induction of polyfunctional HIVconsv-specific CD4+ and CD8+ T cell responses.(a) Box and whisker plot showing background-subtracted CD154/IFN-γ/TNF-α/IL2-positive CD4+ T cell responses to HIVconsv pools stratified by dose (* = p < 0.05, Mann Whitney test). Relationship between CD8+ T cell antiviral activity (% inhibition) and frequencies of CD8+ T cells with either (b) CD107+IFN-γ+TNF-α+/- or (c) CD107-IFNγ+TNFα+/- phenotypes. Pearson R correlation analysis.

The phenotype of HIVconsv-specific CD4+ and CD8+ T cell responses was assessed by intracellular cytokine staining in all volunteers at time points corresponding to their peak Elispot responses. No significant differences in magnitude or phenotype were found between CD8+ T cell responses in placebos, low- or high-dose vaccinees. There was, however, a significantly higher frequency of polyfunctional CD4+ T cells in the high dose vaccines (Figure 6(a)). Interestingly, per cent inhibition was positively correlated with the frequency of HIVconsv-specific CD8+ CD107+ IFN-γ+ TNF-α +/- T cells (r = 0.57, p = 0.01) but not with the frequency of CD8+ CD107- IFN-γ+ TNF-α +/- T cells (r = 0.01 p = 0.94) (Figure 6(b,c).

### Effect of MVA.HIVconsv vaccinations on the HIV-1 reservoir

**Figure 7.**
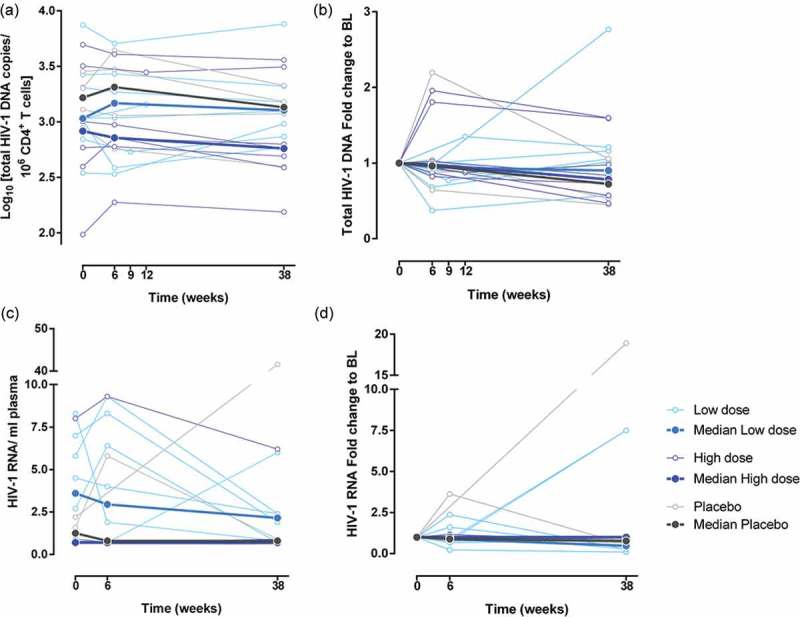
Quantification of the HIV reservoir following vaccination with MVA.HIVconsv.(a) Total HIV-1 DNA (copies/million CD4+ T cells) was measured by quantitative ddPCR in trial participants at baseline (BL), week 6* and week 38. (b) Fold change of total HIV-1 DNA to baseline. (c) Ultrasensitive viral load (HIV-1 RNA/ml plasma) was measured by Abbott Real-Time HIV-1 assay at baseline, week 6 and week 38. (d) Fold change of ultrasensitive viral load to baseline. *In three volunteers total HIV-1 DNA was measured at week 9 or week 12 rather than week 6 due to sample availability, and there was no plasma sample at week 6 for one patient of the placebo group. Dark lines indicate median values for each group and light lines indicate individual values.

Using the upper end of the reported cutoff for detection of HIV-1 DNA by ddPCR (2 copies/10^6^ cells), HIV-1 DNA was detected in all study subjects at all time-points tested (medians 1065, 1014 and 1182 copies/10^6^ CD4+ T cells at weeks 0, 6 and 38 respectively; Figure 7(a)). Copies of HIV-1 DNA/10^6^ CD4+ T cells at baseline did not correlate with CD4+ T cell nadir (r = 0.42, p = 0.15), CD4+ cell count (r = −0.23, p = 0.33), interval between diagnosis and initiation of treatment (r = 0.17, p = 0.54) or duration of ART. There was no change from baseline in the size of the reservoir at week 6 or week 38 post-vaccination in the vaccinees as a whole (medians 1032, 944 and 960 copies HIV-1 DNA/10^6^ CD4+ T cells, p = 0.77; Friedman test) or placebos (medians 1652, 2058 and 1351 copies HIV-1 DNA/10^6^ CD4+ T cells, p = 0.65; Friedman test; Figure 7(a)). Stratification of vaccinees into low- and high-dose groups also did not reveal any significant changes post-vaccination (weeks 0, 6 and 38 medians for low-dose 1074, 1260 and 1272 copies HIV-1 DNA/10^6^ CD4+ T cells, p = 0.65; and high-dose 827, 721 and 577 copies HIV-1 DNA/10^6^ CD4+ T cells, p = 0.23; Friedman test). When HIV-1 DNA was expressed as a ratio of week 6 or week 8 to baseline values, this confirmed that there was no significant change during the study (Figure 7(b)). We detected residual plasma viremia in 47% of the patients, with a range limit of detection (LOD) of 0.6–0.9 copies HIV-1 RNA/ml of plasma (Figure 7(c)). We observed no changes from baseline in the ultrasensitive viral load at weeks 6 or 38 post-vaccination in any group (low-dose, p = 0.68 and 0.12 respectively; high-dose, p = 0.32 and 0.32 respectively; placebo, p = 0.32 and 0.53, respectively; paired Prentice-Wilcoxon test; Figure 7(d)).

## Discussion

We investigated the immunogenicity of a conserved region immunogen, HIVconsv, for the first time in HIV-1 infected individuals on ART. A homologous prime-boost regimen of three MVA.HIVconsv vaccinations was safe and well tolerated. Interferon-γ T cell responses to conserved epitopes, many of which are subdominant in natural infection, were increased after vaccination. However, the magnitude of the change was not significantly greater in vaccinees than placebos, due to an unanticipated level of variation in responses over time in the latter. By contrast, responses to non-HIV-1 antigens remained constant during the study period in vaccinees and placebos. This highlights the natural intra-individual variation in HIV-specific T cell responses in long-term ART-treated patients, which has not been extensively reported before [26]. Furthermore, mapping enabled us to detect a large number of responses to viral epitopes that are infrequently recognized in natural infection, as they were restricted by HLA alleles other than that had been defined previously. This indicates that the HIVconsv immunogen was re-directing responses to subdominant epitopes but as we did not map T cell responses to the entire HIV proteome we cannot prove this point.

These could be attractive targets for more potent immunotherapeutic approaches to purge reactivated CD4+ T cells in which “CTL-resistant” viruses predominate [27]. More potent T cell responses might be elicited using heterologous vaccination regimens comprising a replication-defective chimpanzee adenovirus vector prime followed by MVA boost, as we have reported previously in a trial in healthy HIV-uninfected individuals [28]. Preliminary data indicate that this approach is similarly immunogenic in HIV-positive patients initiating ART during primary infection [29].

In contrast to the lack of effect on IFN-γ responses, vaccination with MVA.HIVconsv did appear to enhance the antiviral inhibitory capacity of CD8+ T cells as there was a significant increase in the subjects receiving the higher dose (2.2 × 10^8^ pfu). Although there was also variation in the placebos, this was not statistically significant. The level of antiviral activity was strongly correlated with the frequency of polyfunctional CD8+ T cells with lytic potential, indicated by upregulation of CD107. The inhibitory activity was still below levels that we have observed in individuals who spontaneously control HIV-1, therefore, these results need to be interpreted with caution [15,30]. The modest responses in the patients studied here may reflect irreversibly compromised CD4+ T cell helper function: although we could detect a significantly higher frequency of polyfunctional CD4+ T cells in the high dose vaccinees, which indicates a degree of T helper function, such responses might be better preserved if ART had been started earlier. This issue has since been addressed in another clinical trial [29].

We reasoned that therapeutic vaccination with MVA.HIVconsv could reduce the size of reservoir through reactivation of latently infected cells [31], for example, via the TLR1/2 signalling pathway [32,33]. In addition, re-expansion of HIV-1-specific CD8+ T cell populations could accelerate the clearance of CD4+ T cells that express cognate epitopes at sufficient levels for immune recognition, whether reactivated or resting [34]. In this study, HIV-1 reservoir size and residual plasma viremia, as measured by total HIV-1 DNA copies in CD4+ T cells and ultrasensitive viral load respectively, did not change significantly after vaccination. While it is possible that viral blips may have occurred in the intervals between vaccination and blood samples, the data suggest that recombinant MVA vaccination alone does not reactivate latent HIV-1 nor induce T cell responses of sufficient potency to perturb the reservoir. This is consistent with another study investigating the effects of an MVA-vectored vaccine encoding HIV-1 Gag [8]. A similar approach involving a DNA /recombinant human adenovirus type 5 prime-boost HIV-1 vaccination strategy in chronically infected patients receiving intensified ART also failed to increase HIV-1 expression or reduce the size of the reservoir [35]. These findings indicate that HIV latency reversal must be synergized with killing by vaccine-induced T cells. T cells must also be able to recognize the epitopes generated by reactivated cells during a short window and ideally target regions of vulnerability that are known to lead to loss of viral fitness upon CD8+ T cell attack *in vivo* [36]. Some of these regions were not included in the HIVconsv immunogen because they were not highly conserved; refinement of the immunogen design may be necessary to ensure boosting of the most potent CD8+ T cell responses [30,37–40]. Analysis of the HLA class I-associated peptidome could facilitate this as we have recently shown, using tandem mass spectrometry, that the HLA class I-associated HIV-1 peptidome on primary CD4+ T cells includes a substantial proportion of epitopes that had not been described previously [41].

This study is, to our knowledge, the first to investigate the relationship between the HIV-1 reservoir size and CD8+ T cell viral inhibitory activity in chronic ART-treated patients. These parameters were not correlated before or after vaccination, possibly reflecting the stability of the reservoir after a median 6 years of infection, and the limited capacity of the vaccine to enhance CD8+ T cell viral inhibitory activity.

Our study has some limitations. It was underpowered to discern differences in the magnitude of T cell responses between vaccine and placebo recipients. The extent of natural variation in HIV-specific T cell responses during ART needs to be considered in the design of future studies. Despite randomization, the vaccinees and placebos were not balanced for protective HLA class I alleles, although this does not appear to have influenced the immunogenicity results. Finally, we used a single assay to quantify the HIV-1 reservoir, based on total, rather than integrated HIV-1. We reasoned that unintegrated forms of HIV-1 DNA make only a small contribution to the size of the viral reservoir after 1 year of ART [42]. Furthermore, total HIV-1 DNA was predictive of time to viral rebound during a supervised therapy interruption in patients treated with ART during primary infection [43].

## Conclusions

Our study highlights the need for further optimization of therapeutic vaccines for inclusion in HIV-1 eradication strategies. In addition, the high degree of variability in HIV-specific IFN-γ T cell responses during long-term ART needs to be considered in clinical trial design; testing of candidate immunological endpoints that are predictive of changes in the viral reservoir is a critical component of such studies.

## Notes

To access the supplementary material to this article please see Supplementary Files under Article Tools online.
